# Store Type and Demographic Influence on the Availability and Price of Healthful Foods, Leon County, Florida, 2008

**Published:** 2011-10-15

**Authors:** Angela F. Leone, Jung Sun Lee, Samantha Rigby, Hilda Kurtz, Mary Ann Johnson, Connie Betterley, Sohyun Park

**Affiliations:** University of Georgia, Athens, Georgia; Department of Foods and Nutrition, University of Georgia; University of Georgia, Athens, Georgia; University of Georgia, Athens, Georgia; University of Georgia, Athens, Georgia; Florida Department of Health, Tallahassee, Florida; Centers for Disease Control and Prevention, Atlanta, Georgia

## Abstract

**Introduction:**

The availability of healthful foods varies by neighborhood. We examined the availability and price of more healthful foods by store type, neighborhood income level, and racial composition in a community with high rates of diet-related illness and death.

**Methods:**

We used the modified Nutrition Environment Measures Survey in Stores to conduct this cross-sectional study in 2008. We surveyed 73 stores (29% supermarkets, 11% grocery stores, and 60% convenience stores) in Leon County, Florida. We analyzed the price and availability of foods defined by the 2005 Dietary Guidelines for Americans as "food groups to encourage." We used descriptive statistics, *t* tests, analysis of variance, and χ^2^ tests in the analysis.

**Results:**

Measures of availability for all more healthful foods differed by store type (*P* < .001). Overall, supermarkets provided the lowest price for most fresh fruits and vegetables, low-fat milk, and whole-wheat bread. Availability of 10 of the 20 fruits and vegetables surveyed, shelf space devoted to low-fat milk, and varieties of whole-wheat bread differed by neighborhood income level (*P* < .05), but no trends were seen for the availability or price of more healthful foods by neighborhood racial composition.

**Conclusions:**

Store type affects the availability and price of more healthful foods. In particular, people without access to supermarkets may have limited ability to purchase healthful foods. Nutrition environment studies such as this one can be used to encourage improvements in neighborhoods that lack adequate access to affordable, healthful food, such as advocating for large retail stores, farmer's markets, and community gardens in disadvantaged neighborhoods.

## Introduction

The 2010 Dietary Guidelines for Americans define more healthful foods as "food groups to encourage," including fruits, vegetables, low-fat milk products, and whole-grain products (1). Although consumption of these foods is recommended to promote health and prevent chronic diseases, studies have found that most Americans do not meet these recommendations (2-4). People who do not consume a nutritious diet are more likely to develop diabetes, cardiovascular disease, obesity, and certain cancers (1,5-7).

The decision to purchase and consume more healthful foods is influenced by personal and environmental factors. The community or consumer nutrition environment has been identified as a priority area of research. The community nutrition environment is the type, location, and accessibility of food stores, and it is described by the availability, price, and quality of food in food stores (8). Previous studies have suggested that understanding the community nutrition environment could provide insight to barriers that may influence dietary behavior (9-11). The relationship of the consumer nutrition environment to more healthful foods is poorly understood. Despite significant interest in consumer nutrition environment research, little progress has been made to devise a reliable and valid tool to be used across all consumer nutrition environment studies (12).

The purpose of this study was to evaluate the consumer nutrition environment of Leon County, Florida, a community with high rates of diet-related illness and death. We used the validated Nutrition Environment Measures Survey in Stores (NEMS-S) to identify potential barriers that some residents may have to accessing healthy, affordable food. We examined the availability and price of more healthful foods by store type, neighborhood income level, and racial composition.

## Methods

We analyzed the price and availability of foods defined by the 2005 Dietary Guidelines for Americans as "food groups to encourage." We used data from the Leon County Nutrition Environment Measures Project, which was initiated, designed, and implemented in 2008 by Florida Department of Health administrators. The study used the most reliable and valid consumer environment measuring tool available to conduct this research. Institutional review board approval was not required for this study because human subjects were not involved.

### Study setting

Leon County is in the panhandle area of Florida. According to 2000 US Census data, the county population was 239,452. The racial composition of the county was 66.4% white, 29.1% black, and 4.5% other race. Approximately 18.2% of residents lived below the federal poverty level, which was higher than the state average of 12.5% (13). Leon County residents have higher rates of childhood obesity and diet-related deaths than do residents of most Florida counties (14).

### Neighborhood characteristics

Census tracts were used as proxies for neighborhoods (15). Using methods similar to those used in previous studies, we dichotomized the 48 census tracts into high- (n = 24) and low-income (n = 24) groups based on the percentage of households living below the federal poverty level in each census tract (16,17) and classified the census tracts into 3 racial groups based on criteria of a previous food environment study (16): predominantly white (<20% of the population was black, n = 11), predominantly black (>80% of the population was black, n = 6), or racially mixed (20%-80% of the population was black, n = 31).

### Store sampling

We obtained a list of supermarkets, grocery stores, and convenience stores from the Florida Department of Agriculture and Consumer Services. Because no standardized definition and classification of food stores has been consistently used in previous nutrition environment studies, we classified stores according to the Department of Agriculture and Consumer Services Florida Administrative Code (18). Food stores are defined on the basis of total square footage, the number of cash registers, and the amount of food processing and service. Stores were then geocoded to census tracts. We used the Florida Community Health Assessment Resource Tool Set to obtain poverty data for each census tract (19). A convenience sampling of the stores was designed to select various store types from each census tract. If possible, a supermarket in each census tract was surveyed. If more than 1 supermarket was available, a chain other than Publix Super Markets was selected to increase the diversity of the sample. If no supermarkets were present, 1 grocery store and 1 convenience store were surveyed. If no supermarkets or grocery stores were found, 2 convenience stores were surveyed. If more than 1 grocery or convenience store was available, stores were randomly selected. This process yielded 65 stores.

Additional supermarket, grocery store, and convenience store sampling was conducted to ensure that stores from the highest- and lowest-income neighborhoods were included. Starting with the census tract with the highest poverty rate, the first census tract that had at least 1 supermarket and more than 2 convenience stores was identified, and all stores within that census tract were surveyed. The same procedure was followed for the census tract with the lowest poverty rate. This criterion yielded 13 more stores, for a total of 78 stores. Five stores were excluded because they did not meet food store definitions or because the store was in an unsafe neighborhood, which yielded a final sample size of 73 stores (29% supermarkets, 11% grocery stores, and 60% convenience stores). Safety was subjectively assessed by 2 observers. The total number of stores sampled is 25% of all stores in Leon County, 100% of all supermarkets, 18% of all grocery stores, and 59% of all convenience stores.

### Food store survey tool

A modified NEMS-S was used to collect data for this study (8). The NEMS-S tool surveys 11 different measures : milk, fruit, vegetables, ground beef and meat alternatives, hot dogs, frozen dinners, baked goods, convenience store and grocery store beverages, bread, baked chips, and baked goods. We modified the survey's fruit and vegetable measures to add items that may be more commonly purchased by low-income people (eg, items commonly found on the Thrifty Food Plan, 1 of 4 USDA plans specifying foods and amounts of foods to provide adequate nutrition and items available in convenience stores). The availability and price of canned fruit cocktail and canned carrots were added to the fruit and vegetable measures, as were canned and frozen produce.

Florida health department administrators attended a 3-day NEMS-S training course conducted by the Emory University researchers who developed and validated the original NEMS-S. The administrators used the modified NEMS-S to conduct a pilot test in 4 stores. After pilot testing, health administrators revised the modified tool and consulted Emory researchers before finalizing the modified tool.

### Survey procedures

Two trained raters surveyed each store between January and March 2008. All store survey protocols followed the original NEMS-S protocol. After the 2 raters completed surveying stores, they compared their scores. When there were discrepancies, raters went back to the store to verify the information.

Availability of all items was recorded by bubbling in yes or no on the survey next to the preferred brand of each item. If the preferred item to be surveyed was unavailable (eg, Red Delicious apples), a similar alternate item was written in (eg, Granny Smith apples). Availability of fresh fruits and vegetables was also measured by counting the total number of types of fruits and vegetables in a store and assigning each a maximum score of 10. For milk availability, shelf space for low-fat milk (skim and 1%) and whole milk in pint, quart, half-gallon, and gallon sizes was measured. Shelf space was measured by counting the total number of available columns  of low-fat and whole-fat milk for each carton size. These numbers were used to calculate the total inches of shelf space devoted to low-fat and whole-fat milk. The availability of whole-wheat bread was also measured by recording the number of different brands and types of whole-wheat and whole-grain bread in a store.

### Price

The lowest price was recorded for all food items. Sale prices were recorded if they were the only prices available and the regular price could not be calculated from the sale price. The price of fruits and vegetables was recorded by piece or by pound. To minimize potential bias, price data for each fruit and vegetable were converted to the unit that was most commonly recorded for that item. The US Department of Agriculture (USDA) nutrient database was used to convert the price of produce from 1 unit to another (eg, 3 medium apples equals 1 pound) (20). The price of milk was recorded for quart and half-gallon sizes. The price of bread was recorded by loaf size (weight in ounces).

### Data analysis

For the purposes of this study, we report the availability and price of all 10 fruits and vegetables on the original NEMS-S, low-fat milk, and whole-wheat bread. Data for each store were entered into the NEMS-S database. Analysis was conducted by Stata data analysis and statistical software, version 10.1 (StataCorp LP, College Station, Texas). Descriptive analysis was conducted to describe the availability and price of each of the food measures. We used *t* tests and analysis of variance tests to compare the continuous availability and price measures of more healthful foods between the 2 neighborhood income-level groups and between the 3 store types and neighborhood race groups, respectively. Fisher's exact tests or χ^2^ tests were used to compare the categorical availability measures by store type and neighborhood characteristics. Nonparametric tests were also used to examine the differences in the price measures by store type and neighborhood characteristics, but the tests provided similar results. The potential interaction between store type and neighborhood characteristics on the continuous availability and price measures were examined by using analysis of variance tests. Significance was set at *P* < .05.

## Results

We analyzed the distribution and percentage of store types included in this study by neighborhood income level and racial composition (Table). Nearly twice as many convenience stores and 7 times more grocery stores were surveyed in low-income neighborhoods than in high-income neighborhoods. Most stores surveyed (64%) were in a mixed-race neighborhood (n = 47). The smallest number of stores was selected from predominantly black neighborhoods; 75% of them were convenience stores.

More than three-quarters of stores surveyed in predominantly white neighborhoods were in high-income neighborhoods. All 8 stores surveyed in predominantly black neighborhoods were in a low-income neighborhood.

### Availability by store type

The availability of all 10 fruits and all 10 vegetables was significantly different by store type (*P* < .001). Four of the fruits were not available in grocery stores and 6 of the fruits were not available in convenience stores, respectively (Figure). All 10 vegetables were available in supermarkets, but none were available in convenience stores.

**Figure.**
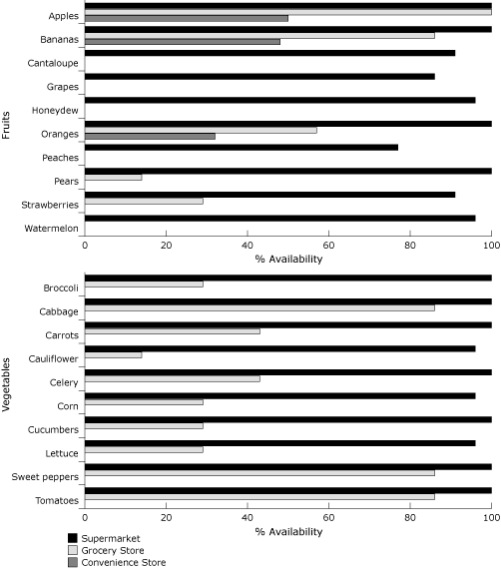
Availability of fruits and vegetables by store type, Leon County, Florida, 2008.

**Table:** 

**Fruit**	**Supermarket**	**Grocery Store**	**Convenience Store**
	**Mean, %**		
Apples	100	100	50
Bananas	100	86	48
Cantaloupe	91	0	0
Grapes	86	0	0
Honeydew	96	0	0
Oranges	100	57	32
Peaches	77	0	0
Pears	100	14	0
Strawberries	91	29	0
Watermelon	96	0	0

**Vegetable**	**Supermarket**	**Grocery Store**	**Convenience Store**
	**Mean, %**		
Broccoli	100	29	0
Cabbage	100	86	0
Carrots	100	43	0
Cauliflower	96	14	0
Celery	100	43	0
Corn	96	29	0
Cucumbers	100	29	0
Lettuce	96	29	0
Sweet peppers	100	86	0
Tomatoes	100	86	0

Supermarkets had the highest fruit availability score (mean [SD], 9.6 [0.93]), on average, compared with grocery stores (3.1 [1.4]) and convenience stores (1.3 [1.3]) (*F* = 325.7, *P* < .001). Supermarkets also had higher vegetable availability scores (10.0), on average, compared with grocery stores (5.1 [3.0]) and convenience stores (0) (*F* = 805.5, *P* < .001).

The availability of low-fat milk differed by store type for both quart and half-gallon sizes (χ^2^ = 23.0 and 23.7, respectively, both *P* < .001). Half-gallon size milk was most commonly available in all 3 store types. All supermarkets (100%) carried low-fat half-gallon milk, compared with 63% of grocery stores and 36% of convenience stores. Supermarkets devoted 52% of shelf space to low-fat milk, compared with 24% in grocery stores and 11% in convenience stores (*F* = 41.6, *P* < .001).

All supermarkets carried whole-wheat bread, compared with 38% of grocery stores and 7% of convenience stores (χ^2^ = 53.0, *P* < .001). Among stores that carried whole-wheat bread, on average, supermarkets carried more varieties (6.0 [0.22]) than did grocery stores (1.1 [2.1]) and convenience stores (0.07 [0.25]) (*F* = 502.7, *P* < .001).

### Price by store type

The prices of 3 fruits (apples, bananas, and oranges, *F* = 25, 3, 189.0, and 15.7, respectively, comparing supermarkets, grocery stores, and convenience stores) and 3 vegetables (cucumbers, lettuce, and peppers, *F* = 8.4, 14.7, and 10.8, respectively, comparing supermarkets and grocery stores) differed by store type: supermarkets provided the lowest price for these fresh fruits and vegetables (*P* < .001). We were able to compare prices for vegetables at supermarkets and grocery stores only, due to the absence of vegetables in convenience stores. The price of low-fat half-gallon size milk (*F* = 26.6, *P* < .001) had a wide price range by store type ($2.22-$5.09). On average, half-gallon and quart size low-fat milk were least expensive in supermarkets and most expensive in convenience stores. When available, whole-wheat bread was least expensive in supermarkets ($2.45 [$0.17]), compared with grocery stores ($2.68 [$0.42]) and convenience stores ($2.62 [$0.12]) but the difference was not significant (*F* = 2.36, *P* = .11).

### Availability by neighborhood characteristics

Availability of each of the 20 fresh produce items was greater in high-income than low-income neighborhoods. Fruit availability scores were significantly higher in high-income than in low-income neighborhood stores (*t* = 2.3, *P* = .02) but did not differ by neighborhood racial composition. Stores in high-income neighborhoods had a larger percentage of shelf space devoted to low-fat milk (33%) than did those in low-income neighborhoods (19%) (*t* = 2.4, *P* = .02). High-income neighborhood stores had more varieties of whole-wheat bread, on average, (2.7 [3.0]) than did low-income neighborhood stores (1.3 [2.4]) (*t* = 2.1, *P* = .04). Availability was not significantly different by neighborhood racial composition.

Neighborhood characteristics were not significantly related to the price of more healthful foods. No significant interactions were found between store type and neighborhood characteristics on the availability and price of healthier food items.

## Discussion

This study documented the availability and price of more healthful foods to better understand the consumer nutrition environment of Leon County, Florida. Our findings suggest that store type is associated with the availability and price of more healthful foods. Neighborhood income level was related to the availability but not the price of some healthful foods.

Greater availability of foods in supermarkets compared with other food stores has been shown (10,21,22). As expected, among surveyed stores, supermarkets had the greatest availability of all more healthful foods, followed by grocery stores and convenience stores.

Several studies have found price differences between store types for various foods (11,23). Few studies have analyzed the price of more healthful foods, but previous studies have consistently found that supermarkets offer more healthful foods at a lower price compared with other food stores (10,24,25). We found significant differences in price by store type for low-fat half-gallon size milk and 6 of 20 fresh fruits and vegetables. These items were significantly less expensive in supermarkets than in grocery stores and convenience stores. Some of the nonsignificant differences in the price of produce may be due to the time of year that the study was conducted, the absence of produce items in grocery and convenience stores, and the use of the USDA nutrient database to roughly estimate price of produce.

Previous consumer nutrition environment studies (16,21,23,25,26) have focused on examining whether the poor and minority neighborhoods have less access to affordable foods and have found inconsistent results. We found no significant differences in the availability and price of more healthful foods by neighborhood racial composition, but neighborhood wealth was associated with significant differences in the availability of 10 of the 20 fruits and vegetable items surveyed, shelf space devoted to low-fat milk, and the availability of varieties of whole-wheat bread. Although it appears that stores in predominantly black and low-income neighborhoods in Leon County provide similar availability and prices of more healthful foods compared with stores in predominantly white and high-income neighborhoods, poor and minority neighborhoods have fewer supermarkets. Supermarkets are distributed fairly equally between high- and low-income level neighborhoods in Leon County, but the distribution of supermarkets by neighborhood racial composition is disproportionate (27). This finding is similar to those of other studies, and this disproportionate distribution may influence the purchasing and consumption of more healthful foods among these populations (24,28).

This study is unique because it was designed and conducted by health department administrators with the intent to make policy changes and interventions in their community. Administrators adopted the best available method by using the validated NEMS-S. Although the study was conducted in a single county at 1 point in time by using a convenience sample of a small number of stores, research has suggested that 1 observation of an area's consumer nutrition environment is sufficient to provide accurate data (29). An additional strength of this study is its focus on the availability and price of more healthful foods that are essential to promoting health and preventing disease and that are the focus of federal dietary guidelines.

This study has many limitations. As with any consumer nutrition environment study, the findings of this study cannot be easily generalized or compared with those of previous studies for the following reasons. First, this study used census tracts to define neighborhoods. Although research has found that residents' definition of a neighborhood is comparable to a census tract, most neighborhoods include parts of at least 2 census tracts (30). Making associations between neighborhoods and their accompanying nutrition environment relies on the assumptions of understanding where people shop for food and the various contexts in which a person's food-related behaviors occur. Second, this study used census tract-level demographic characteristics to determine neighborhood wealth and racial composition. Similar to Morland and Filomena (16), we defined predominantly black as more than 80% of the population. Other studies have defined predominantly black as more than 75% (31) or used more than 50% nonwhite or Hispanic populations or both to define a minority population (26). Third, a range of store type definitions have been used in previous studies. Depending on which source is used, the results of a study may vary. Other potential limitations of the study include the use of the USDA nutrient database to estimate produce prices, inaccurate or unclear data collection resulting from human error, and the convenience sampling that was used to select stores to be audited.

This study suggests that access to supermarkets and more healthful foods varies by neighborhood, which may negatively influence people's eating behavior. By employing the best available tools and method, nutrition environment studies can be used to provide convincing evidence to policy makers, administrators, and consumers that will encourage improvements in neighborhoods that lack adequate access to affordable, healthful food. Examples of such improvements include advocating for large retail stores, farmer's markets, and community gardens in disadvantaged neighborhoods.

## Figures and Tables

**Table.** Distribution and Percentage of Store Types by Neighborhood Income Level and Racial Composition, Leon County, Florida, 2008

Neighborhood Classification	Total Stores (n = 73)	Store Type			

		Supermarkets^a^ (n = 21)	Grocery Stores^b^ (n = 8)	Convenience Stores^c^ (n = 44)

	n (%)			
**Income level** d				
High-income	29 (40)	13 (62)	1 (13)	15 (34)	
Low-income	44 (60)	8 (38)	7 (88)	29 (66)	
**Racial composition** e				
Predominantly white	18 (25)	5 (24)	0	13 (30)	
Mixed race	47 (64)	16 (76)	6 (75)	25 (57)	
Predominantly black	8 (11)	0	2 (25)	6 (14)	

a Retail food stores with 5 or more check-out registers and ≥15,000 sq ft.

b Retail food stores with ≤4 check-out registers and <15,000 sq ft.

c Businesses that sell groceries or motor fuels and may sell coffee or beverages but have no retail food processing, stores with limited food service, or stores with significant food service.

d Census tracts were dichotomized on the basis of the percentage of the population below poverty level. The type of stores included in the study differed significantly by neighborhood income level at *P* < .05 (χ^2^ = 7.37).

e Predominantly white, <20% of population black; mixed race, 20%-80% of population black; predominantly black, >80% of population black.
